# A Cross-Sectional Study of Disparities in Healthcare Transition in Cerebral Palsy

**DOI:** 10.3390/jcm13133759

**Published:** 2024-06-27

**Authors:** Gavin Colquitt, Mario Keko, Haresh D. Rochani, Christopher M. Modlesky, Joshua Vova, Nathalie Linda Maitre

**Affiliations:** 1 Appalachian Institute for Health and Wellness, Beaver College of Health Sciences, Appalachian State University, Boone, NC 28607, USA; 2 Karl E. Peace Center for Biostatistics, Jiann-Ping Hsu College of Public Health, Georgia Southern University, Statesboro, GA 30458, USA; 3 Department of Kinesiology, University of Georgia, Athens, GA 30602, USA; 4 Department of Physical Medicine and Rehabilitation, Children’s Healthcare of Atlanta, Atlanta, GA 30322, USA; 5 Department of Pediatrics, Children’s Healthcare of Atlanta, Atlanta, GA 30322, USA; 6 Department of Pediatrics, Emory University School of Medicine, Atlanta, GA 30322, USA

**Keywords:** cerebral palsy, transition to adult care, healthcare disparities, developmental disabilities, pediatric to adult care transition

## Abstract

**Background:** Cerebral palsy (CP) is the most common physical disability among children, affecting their lifespan. While CP is typically nonprogressive, symptoms can worsen over time. With advancements in healthcare, more children with CP are reaching adulthood, creating a greater demand for adult care. However, a significant lack of adult healthcare providers exists, as CP is predominantly considered a pediatric condition. This study compares the transition experiences of children with CP compared to those with other developmental disabilities (DDs) and typically developing children (TDC). **Methods:** This study utilizes cross-sectional data from the National Survey of Children’s Health (NSCH) from 2016–2020, including 71,973 respondents aged 12–17. Children were categorized into three groups: CP (*n* = 263), DD (*n* = 9460), and TDC (*n* = 36,053). The analysis focused on the receipt of transition services and identified demographic and socioeconomic factors influencing these services. **Results:** Only 9.7% of children with CP received necessary transition services, compared to 19.7% of children with DDs and 19.0% of TDC. Older age, female sex, non-Hispanic white ethnicity, and higher household income were significant predictors of receiving transition services. Children with CP were less likely to have private time with healthcare providers and receive skills development assistance compared to other groups. **Conclusions:** The findings highlight disparities and critical needs for targeted interventions and structured transition programs to improve the transition from pediatric to adult healthcare for children with CP. Addressing disparities in service receipt and ensuring coordinated, continuous care are essential for improving outcomes for children with CP.

## 1. Introduction

Cerebral palsy (CP) is a group of permanent neurological disorders affecting movement and other areas of function that manifest in early childhood and affects the lifespan [1]. It is the most common movement disorder among children, with 1 in 323 American children diagnosed each year and 1.6 in 1000 in high-income countries [2,3]. Although the brain lesions that cause CP are typically defined as nonprogressive, manifestations of CP may worsen over time [4]. With advancements in medicine and healthcare, a growing number of children with CP now reach adulthood [5], and there is a greater demand among adolescents and adults with CP to receive specialized care [6]. However, there is a significant lack of adult providers for these individuals with CP because CP is largely considered a pediatric condition [7].

Although adult providers are scarce, children with CP may also face inadequate transition support from pediatric providers. Most young people with CP do not receive care that follows best-practice guidelines for the transition from pediatric to adult care [8]. This multifaceted process should center around the patient and their continuity of care [9]. Healthcare transitions from adolescence to adulthood involve complex and multidimensional processes, emphasizing the need for structured transition planning, coordinated transfer, and integration into adult care systems. However, most youth with special healthcare needs do not receive adequate support, leading to adverse outcomes such as discontinuity of care with missed diagnoses, increased hospitalizations, and higher healthcare costs [10]. A myriad of inconsistencies in transition practices exist, even among multidisciplinary clinics specializing in care for CP [11]. Pediatric healthcare is typically overseen by a pediatrician who has a comprehensive understanding of the patient’s health and trajectory; a comparable comprehensive understanding provided by an adult physician could be suitable for managing CP in adult patients [12].

Effective coordination of care for adolescents and young adults requiring specialized, complex care significantly reduces hospitalizations and emergency department visits, leading to lower healthcare costs [13]. The Transition Research Programme (UK) determined that the following nine services features were associated with improved outcomes for children with chronic conditions: age-banded clinics; meeting with the adult team before transfer; promotion of health self-efficacy; written transition plan; appropriate parent involvement; key worker assigned; coordinated team; holistic life-skills training; and a transition manager for the clinical team [14]. However, a longitudinal study examining service utilization from 2000 to 2010 found that these services are often lacking when children with CP transition from pediatric to adult care [15].

The lack of focus on transition among children with CP compared to other children with developmental disabilities (DDs) could be because much of the literature has concentrated on a broader group of children with chronic conditions and other developmental disabilities. For example, a systematic review of transition-of-care interventions for youth with disabilities evaluated the effectiveness of various transitional care interventions, highlighting that structured transition programs and case management were generally effective in improving transition-related outcomes such as the continuity of care, patient satisfaction, and health outcomes [9]. Similarly, Colver et al. [15] included a sample of 374 children with type 1 diabetes, CP, and autism spectrum disorders. While there are commonalities in needs among various groups of children with special healthcare needs, CP is a complex disorder that requires the involvement of multiple service providers, highly specialized care, and accurate, specific information for both children and their families. A scoping review of information needs for transition-aged children with CP and their families revealed that individuals with CP, their families, and adult caregivers have specific information requirements as they transition to adulthood [16]. Both young people with CP and their families desired insights into the nature of adult services and the methods of acquiring assistance, and youth with CP expressed a preference for personalized information delivery based on their immediate needs, as opposed to group sessions or paper handouts [16].

There is currently little to no research on transitioning from pediatric to adult care among children with CP from a nationally representative sample in the United States. The National Survey of Children’s Health (NSCH) is a cross-sectional, population-based survey with state- and national-level estimates of multiple factors related to child health. In 2016, the survey began including questions related to the transition experiences of children from pediatric to adult care. This study aims to fill this gap by comparing the transition experiences of children with CP to those of children with other developmental disabilities (DDs) and typically developing children (TDC). We hypothesize that children with CP receive fewer transition services compared to their peers with DDs and TDC. Given the documented challenges and gaps in transition services for children with CP, this study aims to provide evidence that can inform targeted interventions and structured transition programs to improve outcomes for this population.

## 2. Methods

### 2.1. Study Design

This study employs a cross-sectional design, utilizing data from the NSCH conducted between 2016 and 2020. The NSCH is a population-based survey that provides state- and national-level estimates of various factors related to child health. This survey was selected for its comprehensive and representative data on children’s health and transition experiences. The NSCH includes validated questions [17] related to healthcare transition, making it suitable for examining the transition experiences of children with CP, other DDs, and TDC. The survey methodology and questions have been validated in previous studies and are consistent with established practices for health surveys [17].

### 2.2. Participants

Participants included parent respondents to the 2016–2020 (5 years combined) NSCH. This is an annual survey sponsored by the US Department of Health and Human Services, Health Resources and Services Administration (HRSA), and Maternal and Child Health Bureau (MCHB) and is conducted by the US Census Bureau [17]. Age categories were 0–5 years, 6–11 years, and 12–17 years. Children with special healthcare needs and children ages 0–5 were oversampled. This study only examined children in the 12–17 age category because questions related to the receipt of transition services were only asked of children ages 12 years and older. Of the 71,973 responding parents of children aged 12–17 years old, 263 of them reported having a child with CP, 9620 reported having a child with a DD, and 36,053 with a TDC.

### 2.3. Instrumentation

The variables used in this study were gathered from the 2016–2020 NSCH, Family Health Measures and Subgroups and SAS Codebook [18]. The NSCH uses complex survey weighting to adjust for the probability of selection, nonresponse, and demographic variations. These weights ensure that the survey results are representative of the U.S. child population, allowing for accurate national and state-level estimates. Participants completed a validated household pre-survey screener and were asked to complete a child-level survey for a randomly selected child in their household. After the pre-screener survey was completed, a child was selected at random, and parents were asked to complete an age-appropriate topical questionnaire. The survey was administered by mail and online. Most households completed the survey online; however, hard copies were sent to homes if necessary. In addition, two followup notices were mailed to participants to remind them to complete the survey.

### 2.4. Definition of Groups: CP, DDs, TDC

Based on the survey answers, we created three groups from the original 71,973 respondents aged 12–17. Inclusion criteria were based on specific diagnoses to ensure clear and distinct group classifications, while exclusion criteria focused on avoiding overlap between CP and DDs to maintain the integrity of the comparisons. A child was included in the CP group if their parent responded “Yes” to the question, “Has a doctor or other healthcare provider EVER told you that this child has Cerebral Palsy?” A child was placed in the DD group if their parent responded “Yes” at least once to whether a doctor or other healthcare provider ever told them that their child has Down syndrome, brain injury, concussion or head injury, developmental delay, intellectual disability, autism, or autism spectrum disorder (and the disorder is current) while also responding “No” to the question about Cerebral Palsy. The third group of TDC consisted of children whose parents responded “No” to the questions about CP or DDs and a “No” response to whether a doctor or healthcare provider ever told them that their child has epilepsy or seizure disorders, blood disorders, genetic disorders, cystic fibrosis, diabetes, or another chronic condition. Over the five-year period from 2016 to 2020, a total of 263 children in the CP group, 9460 in the DD group, and 36,053 in the TDC group participated in the annual survey, with different families sampled each year.

### 2.5. Transition to Adult Healthcare

We examined receipts of any transition-related services, defined as one of the following: (1) a private conversation between the child and the doctor occurred during the latest preventive check-up, without an adult in the room; (2) a discussion was had, if needed, regarding the transition to adult care; and (3) there was evident active collaboration between the doctor and the child to enhance skills and understand healthcare changes. Parents had to indicate that their child received all three services to meet the criteria for *receipt* of transition services.

### 2.6. Covariates

Demographic variables such as the child’s age, sex, race, ethnicity, and household income expressed in terms of federal poverty level (FPL) were included in the statistical models as covariates. Children between the ages of 12 and 17 were the focus of this study. The child’s age, measured in years, was treated as a continuous variable and controlled for in the analysis. Sex was divided into “female” or “male” categories. Ethnicity was divided into four categories: “non-Hispanic white”, “non-Hispanic black”, “Hispanic”, or “Other”. Finally, the child’s household income was reported in comparison to the federal poverty level and was split into “0–199% FPL” and “200% FPL or above”, with children at or below the 199% FPL qualifying for state children’s health insurance program benefits [18].

### 2.7. Data Analysis

After ensuring the consistency of the survey questions of interest, the data from each year were combined following the NSCH Guide to Multi-Year Analysis [19]. Three groups were created from the final dataset: children with CP, children with a DD, and TDC. Descriptive statistics were generated for the frequency of each variable of interest by group (children with CP vs. children with other DDs vs. TDC). Data preparation and statistical analysis were performed using SAS software, Version 9.4 of the SAS System for Windows. Chi-square tests and ANOVA were used to measure the associations between the variables of interest and groups. Binary logistic regression was used to estimate the effect of the presence of CP and DDs on receiving the services necessary for the transition to adult healthcare. The analysis also addressed the issue of missing data in several key variables using multiple imputation. For the variable “received services needed to transition to adult healthcare”, data were missing for 299 respondents (0.64%). The variable “children who had time alone with healthcare provider” had missing data for 769 respondents (1.66%). A larger proportion of missing data was observed for the variable “provider worked with child to gain skills to manage health/healthcare or understand healthcare changes at age 18”, with 6290 respondents (13.55%) missing this information. The variable “provider discussed shift to adult healthcare providers” had the highest proportion of missing data, with 20,648 respondents (44.48%) missing this information. The impact of these missing datapoints on the overall results was considered, and appropriate statistical methods were employed to ensure the robustness of the findings. In our data analysis, we checked for linearity by examining the relationship between continuous predictors and the logit of the outcome, ensured the independence of errors by utilizing the design of the NSCH, and examined the variance inflation factor (VIF) for each predictor to confirm the absence of multicollinearity. We addressed the issue of missing data in several key variables using multiple imputation through Full Conditional Specification (FCS), the corresponding procedures ‘PROC MI’ in SAS Software [20]. This was followed by fitting the logistic regression models of interest and using ‘PROC MI’ to pool across the imputed datasets (number of imputations = 30) the regression estimates of interest.

## 3. Results

Table 1 presents the characteristics of children with CP, children with DDs, and TDC. The average age across all groups was similar, with no significant differences noted. However, there were notable disparities in other characteristics. The proportion of males was higher among children with DDs (64.5%) compared to children with CP (54.0%) and TDC (48.6%), with children with CP showing significantly lower odds (OR 0.65, 95% CI 0.51–0.83) of being male compared to children with DDs.

Ethnicity also varied significantly among groups. Non-Hispanic black children were more prevalent among children with CP (9.5%) compared to children with DDs (5.8%) and TDC (6.1%), with children with CP having higher odds (OR 1.82, 95% CI 1.19–2.80) of being non-Hispanic black compared to children with DDs. Additionally, children with CP were more likely to live at or below 199% of the federal poverty level (FPL) (33.8%) compared to children with DDs (28.6%) and TDC (26.0%), with significant differences observed (OR 1.48, 95% CI 1.10–2.00).

In terms of healthcare transitioning, 9.7% of children with CP were reported as receiving all 3 services necessary for transitioning to adult healthcare, significantly lower than that of children with DDs (19.7%) and TDC (19.0%); children with CP had lower odds (OR 0.44, 95% CI 0.29–0.66) of receiving such services compared to children with DDs. Children with CP were less likely to have private time with healthcare providers (24.1%) compared to children with DDs (47.5%) and TDC (41.7%) (OR 0.35 CP vs. DD, 95% CI 0.26–0.47).

Regarding skill development for managing health, children with CP also had lower odds (OR 0.73, 95% CI 0.57–0.95) compared to children with DDs. However, there were no significant differences in discussing a shift to adult healthcare providers among the groups, with 25.0% of children with CP, 22.7% of children with DDs, and 22.3% of TDC having these discussions.

Table 2 provides a summary of the number and distribution of children within the DD group which totaled 9425. Among these participants, 150 (1.6%) were diagnosed with Down syndrome, 4255 (45.17%) had experienced a brain injury, concussion, or head injury, and 4677 (49.65%) were identified as having developmental delays. Additionally, 900 individuals (9.55%) were reported to have intellectual disabilities, while 2373 participants (25.19%) were diagnosed with autism spectrum disorders.

Table 3 presents the disparities among children with CP, children with DDs, and TDC in receiving the services necessary for transitioning to adult healthcare. Children with CP were 55% less likely to receive these services compared to TDC (AOR 0.45, 95% CI 0.30–0.68, *p* = 0.0004), while there were no statistically significant differences between children with DDs and TDC (AOR 0.99, 95% CI 0.94–1.05, *p* = 0.7878).

Age was a significant factor, with the odds of receiving transition services increasing by 34% for each additional year of age (AOR 1.34, 95% CI 1.32–1.36, *p* < 0.0001). Male children were 9% less likely to receive the necessary services compared to female children (AOR 0.91, 95% CI 0.87–0.95, *p* < 0.0001).

Ethnicity also played a significant role. Non-Hispanic white children were 26% more likely to receive transition services compared to children of other ethnicities (AOR 1.26, 95% CI 1.16–1.36, *p* < 0.0001). However, there were no significant differences for non-Hispanic black children (AOR 0.88, 95% CI 0.79–1.01, *p* = 0.0631) or Hispanic children (AOR 0.94, 95% CI 0.84–1.04, *p* = 0.2262) compared to children of other ethnicities.

Household income, measured in terms of the FPL, was another important factor. Children from households with incomes at 0–199% of the FPL were 14% less likely to receive transition services compared to those from households at or above 400% of the FPL (AOR 0.86, 95% CI 0.81–0.91, *p* < 0.0001). Similarly, children from households with incomes at 200–299% of the FPL were 12% less likely to receive these services (AOR 0.88, 95% CI 0.82–0.95, *p* = 0.0004). There were no significant differences for children from households with incomes at 300–399% of the FPL (AOR 0.95, 95% CI 0.88–1.02, *p* = 0.1338).

## 4. Discussion

The transition from pediatric to adult healthcare is a critical period for children with CP and presents numerous challenges that can significantly impact their quality of life. The findings from this study underscore the complexities and disparities in the transition experiences of children with CP compared to children with DDs and TDC. Children with CP are significantly less likely to receive necessary services that best support or promote a successful transition to adult healthcare. Only 9.7% of the children with CP in this study received these services, which is substantially lower than the rates for children with DDs (19.7%) and TDC (19.0%). Although some guidelines and best practices to support the transition process for children with long-term, chronic conditions [14,15], these do not exist for children with CP in the US. The lower odds of receiving transition services among children with CP highlights the need for targeted interventions to address these gaps in care.

Age was a significant factor in receiving transition services, with older children being more likely to receive such services (AOR 1.34, 95% CI 1.32–1.36). This finding aligns with previous research indicating that transition planning often intensifies as children approach adulthood [21]. However, there were no significant differences in discussing a shift to adult healthcare providers among the groups, with 25.0% of children with CP, 22.7% of children with DDs, and 22.3% of TDC having these discussions. The explanation for only a fraction of children having these discussions is potentially due to the fact that these conversations may occur over three- to four-year periods recommended for transition and not all at once at every visit [10]. This means that during annual visits, not every element may be addressed, except perhaps closer to the transition to an adult provider. According to a clinical report from the American Academy of Pediatrics (AAP) [10], the transition process involves preparation, planning, tracking, and follow-through beginning in early adolescence and continuing into young adulthood, which supports this gradual approach. However, neither the Health Resources and Services Administration’s Maternal and Child Health Bureau nor the AAP have discrete implementation recommendations or toolkits and instead provide general guidance to support physicians in daily practice. Physicians need clear schedules, checklists, and processes that match the AAP recommended primary care visits during the 12–18-year range. Developing a stronger implementation plan would likely help improve this issue for all children, not only those with CP. It is important to note that for TDC, transition discussions may not be as relevant since they are less likely to require regular hospital services; these discussions can still be beneficial for preparing them for independent healthcare management as they approach adulthood. However, there would likely still be a gap for children with CP, requiring a more targeted approach.

The overall low rates of transition service receipt among children with CP suggest that efforts to improve transition planning need to start earlier and be more consistent, as also recommended by Liljenquist et al. [21]. This study also revealed significant disparities based on sex and ethnicity. Male children were less likely to receive transition services compared to female children (AOR 0.91, 95% CI 0.87–0.95), and non-Hispanic white children were more likely to receive these services compared to children of other ethnicities (AOR 1.26, 95% CI 1.16–1.36). These findings suggest that gender and racial biases may influence the receipt of transition services, necessitating policies and practices that promote equity in healthcare access, as also recommended by Bolger et al. [11]. Household income also played a role in the receipt of transition services. Children from lower-income households (0–199% FPL) were significantly less likely to receive transition services compared to those from higher-income households (AOR 0.86, 95% CI 0.81–0.91). This finding indicates that socioeconomic status is a major barrier to accessing necessary healthcare services, which can exacerbate health disparities as these children transition to adulthood [15]. To support improvements in addressing gender and racial biases in the provision of transition services, the implementation of structured processes that ensure equitable access is necessary. Standardized transition protocols may help mitigate biases by providing clear guidelines that healthcare providers must follow. Additionally, these processes should be culturally tailored to reflect the specific needs and contexts of the populations they serve. This involves training healthcare professionals to recognize and address their own biases and to understand the cultural and socioeconomic factors that influence healthcare access and outcomes. Culturally appropriate transition processes, which consider the unique challenges of marginalized groups that are adaptable to various service settings and country-specific healthcare systems, are crucial.

The successful transition from pediatric to adult care is a pivotal milestone with far-reaching implications for the health, well-being, and independence of young people with CP [22]. However, much of the literature has focused on broader groups of children with chronic conditions, which may not fully capture the unique needs of children with CP. CP is a complex disorder requiring multiple service providers and highly specialized care [11]. Additionally, the existing literature on the impact of transition services for children with CP provides varying perspectives. For instance, data from the same study involving children with CP found no significant differences in unmet needs between those who received transition services and those who did not [12]. However, this study did not assess the effectiveness or other aspects of the transition, indicating that a formal transition from one care setting to another may not necessarily imply its effectiveness. Children with CP already arrive at the transition age with specific unmet needs compared to typical adolescents, and these needs have been demonstrated to increase over time [12].

To improve transition outcomes, it is essential to implement structured transition planning that is individualized and coordinated. The Transition Research Programme in the UK identified key service features associated with improved outcomes, such as age-banded clinics, meeting the adult team before transfer, promoting health self-efficacy, having a written transition plan, appropriate parent involvement, and having a key worker and transition manager for the clinical team [14]. However, these services are often not present or partially provided, leaving many young people with CP and their families lacking the support they need [15].

The Ireland-based and UK guidelines for transition services are examples of potential supports that exist to aid the transition process [15]. However, these guidelines are often not implemented consistently, as evidenced by a study showing that few adults with CP report receiving support in accordance with these guidelines [8]. Only one in four adults with CP reported receiving any formal information about the transition process, and less than one in five report receiving an opportunity to meet their new adult providers, life skills training, or self-management support for mental health [8]. In addition to facing potential care issues during the transition from pediatric to adult care, children with CP also experience a significant decline in the use of therapy services after graduating from school [21]. In turn, adults with CP experience worse health outcomes such as higher rates of obesity, hypertension, and depression [23]. As adults with CP age, there is a sharp decrease in their ability to independently perform activities of daily living [23].

A significant barrier in the transition process for children with CP is the lack of coordination and communication between pediatric and adult healthcare providers as the absence of a unified approach often results in fragmented care and confusion [24]. Brandon et al. [6] examined the transition to primary care services by interviewing adults with CP, pediatricians, parents, and primary healthcare physicians in Canada. To overcome the many challenges adults with CP face in accessing health services, the authors recommended fostering ongoing partnerships between pediatric and adult health care providers to ensure seamless care continuity and significantly improve outcomes by establishing connections to team-based primary care services where family physicians, subspecialists, and interprofessional practitioners collaborate on joint care planning. This challenge is identified in the literature because children with CP are accustomed to receiving coordinated care in multidisciplinary specialty clinic settings with various subspecialties, whereas adult care for people with CP is more fragmented and isolated, placing more responsibility on the adult to coordinate their own care [25,26,27].

To effectively address the transition from pediatric to adult care for children with CP, a comprehensive and multidisciplinary approach is essential. Both pediatric and adult care providers must work collaboratively to ensure a seamless transfer of care, emphasizing regular communication and shared care planning. However, this will likely only be realized through the establishment of structured transition programs that involve the patient, family, and a multidisciplinary team of healthcare providers [22]. These programs should also contain features that empower young individuals with CP by involving them early on in their care decisions and enhancing their self-advocacy skills, along with increasing the availability of knowledgeable adult healthcare providers and ensuring their integration into the care team [28]. The review by Levy et al. [9] highlights the effectiveness of structured transition programs and case management in improving transition-related outcomes for youth with disabilities, such as continuity of care, patient satisfaction, and health outcomes.

Approximately one third of children in the DD group had autism spectrum disorder or an intellectual disability. These conditions often receive substantial attention and resources in the US, which may not be the case for other conditions like CP. For example, although CP is the most common childhood physical disability, it was only recently added to the Center for Disease Control and Prevention’s Autism and Developmental Disabilities Monitoring (ADDM) Network [29]. Notably, there has been a significant 20-year disparity in CDC funding between autism spectrum disorder [29], or Fragile X (with a prevalence of 1/7000–11,000, the leading genetic cause of autism spectrum disorder) [30], and CP (with a prevalence on 1/323), with CP receiving no funding. This public health disparity underscores the need for equitable resource allocation across all developmental conditions.

Coyle and Atkinson’s [31] model of perception of vulnerability in medical practice, which views patients with special healthcare needs as needing paternalistic protection, may explain why children are not left alone with their providers or why time alone with the child to develop a healthcare transition plan is not requested. In this study, despite facing similar challenges, children with DDs received more transition services, likely due to better societal integration, whereas children with CP, who often experience more pain, impaired motor function, and communication difficulties, tend to have significantly lower participation rates in society [32]. This disparity in integration and resource allocation must be addressed to improve outcomes for children with CP at a systemic and structural level. This study has several limitations that should be acknowledged. First, the data are based on parent-reported information from the NSCH, which may be subject to recall bias or misreporting.

The cross-sectional nature of the survey limits the ability to establish causality between the variables studied. The missing data for several key variables, such as “received services needed to transition to adult healthcare” and “provider worked with child to gain skills to manage health/healthcare or understand healthcare changes at age 18”, also pose a limitation. Although multiple imputation was used to address this issue, the missing data could still affect the robustness of the findings. There were also limitations based on the specific aspects of the condition of each child. A child was placed in the DD group if their parent reported the condition of Down syndrome, brain injury, concussion or head injury, developmental delay, intellectual disability, autism, or autism spectrum disorder. This did not assess the severity of each condition and, in some cases, was vague. For example, the type of developmental delay was not specified. Lastly, the survey’s reliance on self-administered questionnaires, either online or via mail, may exclude households with limited access to the internet or lower literacy levels, potentially introducing a selection bias. These limitations highlight the need for caution when interpreting the results and underscore the importance of further research to validate and extend these findings. In addition, potential biases, such as recall bias from parent-reported data and the limitations inherent in a cross-sectional study design, could affect the accuracy and generalizability of our findings, potentially leading to either an overestimation or underestimation of the receipt of transition services. The generalizability of our study results is supported by the use of the NSCH, which provides a large, nationally representative sample of children in the US. This comprehensive dataset includes diverse demographic and socioeconomic groups, enhancing the external validity of our findings. However, the reliance on parent-reported data and the cross-sectional nature of the survey may limit the applicability of our results to other contexts or populations. Despite these limitations, the consistency of our findings with the existing literature suggests that the identified disparities in transition services for children with CP are likely reflective of broader trends and challenges faced by this population across the US.

Gaps highlighted here constitue a call-to-arms for guidelines allowing children with CP to have comprehensive and evidence-based transition-to-adulthood services, such as those existing for far less common neurological conditions [33]; guidelines should explicitly account for healthcare disparities stemming from adverse social determinants of health, more prevalent in CP than in other DDs. These guidelines must address the unique medical, social, and psychological needs of individuals with CP to ensure they receive continuous, high-quality care throughout their lives. Developing a standardized approach to transition care that includes early and consistent planning, equitable access to services and the empowerment of young individuals through self-advocacy training are essential. Utilizing frameworks and checklists, such as those provided by the Got Transition initiative (https://www.gottransition.org, accessed on 16 June 2024), can standardize transition practices and ensure comprehensive care. Furthermore, incorporating culturally appropriate transition processes tailored to specific service and country contexts can address racial and gender biases in service provision. Engaging with the lived experiences of individuals with CP, as highlighted in recent studies, can inform and refine local transition approaches to better meet the unique needs of this population [34]. Additionally, integrating adult healthcare providers knowledgeable about CP (or upskilling adult providers) into multidisciplinary care teams will help mitigate many of the barriers identified in this study. Policymakers and healthcare organizations must prioritize the establishment of these guidelines to bridge the existing gaps and ensure that all young people with CP have the support they need to thrive in adulthood. Only through a concerted effort to address these disparities can we hope to improve the long-term health outcomes and overall quality of life for individuals with CP.

## Figures and Tables

**Table 1:** Descriptive characteristics and odds ratios for transition-to-adult-healthcare services among children with cerebral palsy, developmental delay, and typically developing children.

	No. (%)				
Descriptive characteristics	CP	DD	TDC	CP vs. DD Odds Ratio	CP vs. DD + TDC Odds Ratio
	(*n* = 263)	(*n* = 9420)	(*n* = 36,053)	(95% CI)	(95% CI)
Age					
Mean ± SD	14.8 ± 1.8	14.9 ± 1.7	14.7 ± 1.70	0.98 (0.91, 1.05)	1.03 (0.96, 1.11)
Sex					
Male	142 (54.0)	6072 (64.5)	17,839 (48.6)	0.65 (0.51, 0.83) *	1.09 (0.86, 1.39)
Female	121 (46.0)	3448 (35.5)	18,896 (51.4)	reference	reference
Ethnic group					
Non-Hispanic white	178 (67.7)	7097 (75.3)	25,506 (69.4)	reference	reference
Non-Hispanic black	25 (9.5)	548 (5.8)	2223 (6.1)	1.82 (1.19, 2.80) *	1.65 (1.09, 2.52) *
Hispanic	29 (11.0)	888 (9.4)	4449 (12.1)	1.30 (0.87, 1.94)	1.00 (0.67, 1.48)
Other	31 (11.8)	887 (9.4)	4557 (12.4)	1.39 (0.95, 2.05)	1.04 (0.71, 1.53)
Poverty level					
0–199%	89 (33.8)	2695 (28.6)	9560 (26.0)	1.48 (1.10, 2.00) *	1.65 (1.23, 2.22) *
200–299%	47 (17.9)	1443 (15.3)	5778 (15.7)	1.46 (1.02, 2.09) *	1.48 (1.04, 2.11) *
300–399%	39 (14.8)	1346 (14.3)	5330 (14.5)	1.30 (0.88, 1.90)	1.33 (0.91, 1.94)
≥400%	88 (33.5)	3963 (41.8)	16,067 (43.7)	reference	reference
Received Transition Services					
Yes	25 (9.7)	1842 (19.7)	6945 (19.0)	0.44 (0.29, 0.66) *	0.45 (0.30, 0.68) *
No	233 (90.3)	7519 (80.3)	29,555 (81.0)	reference	reference
Children who had time alone with healthcare provider					
Yes	61 (24.1)	4403 (47.5)	15,060 (41.7)	0.35 (0.26, 0.47) *	0.42 (0.32, 0.57) *
No	192 (75.9)	4868 (52.5)	21,065 (58.3)	reference	reference
Provider worked with child to gain skills to manage health/healthcare or understand healthcare changes at age 18					
Yes	139 (57.0)	5441 (64.4)	19,702 (62.7)	0.73 (0.57, 0.95) *	0.78 (0.60, 1.00)
No	105 (43.0)	3006 (35.6)	11,735 (37.3)	reference	reference
Provider discussed shift to adult healthcare providers					
Yes	50 (25.0)	1371 (22.7)	4348 (22.3)	1.13 (0.82, 1.57)	1.16 (0.94, 1.60)
No	150 (75.0)	4658 (77.3)	15,193 (77.7)	reference	reference

* *p* < 0.001.

**Table 2:** Prevalence of developmental and neurological conditions among children with developmental disabilities.

Condition	N (%)
Down Syndrome	150 (1.6)
Brain Injury, Concussion, or Head Injury	4255 (45.17)
Developmental Delay	4677 (49.65)
Intellectual Disability	900 (9.55)
Autism Spectrum Disorder	2373 (25.19)

**Table 3:** Differences in receipt of transition services between children with cerebral palsy, developmental disabilities, and typically developing children.

Variable	AOR	(95% CI AOR)	*p* Value	Likelihood Estimate
Group ^a^				
CP	0.45 ^e^	(0.30, 0.68)	0.0004 ^e^	
DD	0.99	(0.94, 1.05)	0.7878 ^e^	55% less likely
Age	1.34 ^e^	(1.32, 1.36)	<0.0001	34% more likely
Sex ^b^				
Male	0.91 ^e^	(0.87, 0.95)	<0.0001	9% less likely
Ethnicity ^c^				
Black, non-Hispanic	0.88	(0.79, 1.01)	0.0631	
Hispanic	0.94	(0.84, 1.04)	0.2262	
White, non-Hispanic	1.26 ^e^	(1.16, 1.36)	<0.0001	26% more likely
Household income in terms of FPL ^d^				
0–199%	0.86 ^e^	(0.81, 0.91)	<0.0001	14% less likely
200–299%	0.88 ^e^	(0.82, 0.95)	0.0004	12% less likely
300–399%	0.95	(0.88, 1.02)	0.1338	

AOR—adjusted odds ratio; CI—confidence interval; FPL—Federal Poverty Level; ^a^ reference category is TDC; ^b^ reference category is Female; ^c^ reference category is Others; ^d^ reference category is 400% FLP or above; ^e^ HOCHBERG adjusted *p*-value controlling for multiple comparisons.

## Data Availability

All data for this study is available upon request from the Child and Health Measurement Initiative at https://www.childhealthdata.org.

## References

[1] Rosenbaum P., Paneth N., Leviton A., Goldstein M., Bax M., Damiano D., Dan B., Jacobsson B. (2007). A report: The definition and classification of cerebral palsy April 2006. Dev. Med. Child Neurol..

[2] Christensen D., Braun K.V.N., Doernberg N.S., Maenner M.J., Arneson C.L., Durkin M.S., E Benedict R., Kirby R.S., Wingate M.S., Fitzgerald R. (2014). Prevalence of cerebral palsy co-occurring autism spectrum disorders and motor functioning—Autism and Developmental Disabilities Monitoring Network, USA, 2008. Dev. Med. Child Neurol..

[3] McIntyre S., Goldsmith S., Webb A., Ehlinger V., Hollung S.J., McConnell K., Arnaud C., Smithers-Sheedy H., Oskoui M., Khandaker G. (2022). Global prevalence of cerebral palsy: A systematic analysis. Dev. Med. Child Neurol..

[4] Lin J.-P., Lumsden D.E., Gimeno H., Kaminska M. (2014). The impact and prognosis for dystonia in childhood including dystonic cerebral palsy: A clinical and demographic tertiary cohort study. J. Neurol. Neurosurg. Psychiatry.

[5] Blair E., Langdon K., McIntyre S., Lawrence D., Watson L. (2019). Survival and mortality in cerebral palsy: Observations to the sixth decade from a data linkage study of a total population register and National Death Index. BMC Neurol..

[6] Brandon E., Ballantyne M., Penner M., Lauzon A., McCarvill E. (2019). Accessing primary healthcare services for transition-aged young adults with cerebral palsy: Perspectives of young adults, parents, and physicians. J. Transit. Med..

[7] Peterson M.D., Hurvitz E.A. (2021). Cerebral palsy grows up. Mayo Clin. Proc..

[8] Ryan J.M., Walsh M., Owens M., Byrne M., Kroll T., Hensey O., Kerr C., Norris M., Walsh A., Lavelle G. (2023). Transition to adult services experienced by young people with cerebral palsy: A cross-sectional study. Dev. Med. Child Neurol..

[9] Levy B.B., Song J.Z., Luong D., Perrier L., Bayley M.T., Andrew G., Arbour-Nicitopoulos K., Chan B., Curran C.J., Dimitropoulos G. (2020). Transitional care interventions for youth with disabilities: A systematic review. Pediatrics.

[10] White P.H., Cooley W.C., Boudreau A.D.A., Cyr M., Davis B.E., Dreyfus D.E., Forlenza E., Friedland A., Greenlee C., Mann M. (2018). Supporting the healthcare transition from adolescence to adulthood in the medical home. Pediatrics.

[11] Bolger A., Vargus-Adams J., McMahon M. (2017). Transition of care in adolescents with cerebral palsy: A survey of current practices. PM&R.

[12] Solanke F., Colver A., McConachie H., Transition Collaborative Group (2018). Are the health needs of young people with cerebral palsy met during transition from child to adult healthcare?. Child Care Health Dev..

[13] Maeng D.D., Snyder S.R., Davis T.W., Tomcavage J.F. (2017). Impact of a complex care management model on cost and utilization among adolescents and young adults with special care and health needs. Popul. Health Manag..

[14] Colver A., McConachie H., Le Couteur A., Dovey-Pearce G., Mann K.D., McDonagh J.E., Pearce M.S., Vale L., Merrick H., Parr J.R. (2018). A longitudinal, observational study of the features of transitional healthcare associated with better outcomes for young people with long-term conditions. BMC Med..

[15] Colver A., Pearse R., Watson R.M., Fay M., Rapley T., Mann K.D., Le Couteur A., Parr J.R., McConachie H. (2018). How well do services for young people with long-term conditions deliver features proposed to improve transition?. BMC Health Serv. Res..

[16] Freeman M., Stewart D., Cunningham C.E., Gorter J.W. (2018). Information needs of young people with cerebral palsy and their families during the transition to adulthood: A scoping review. J. Transit. Med..

[17] United States Census Bureau (2021). 2020 National Survey of Children’s Health Methodology Report.

[18] Child and Adolescent Health Measurement Initiative (CAHMI) (2022). 2020 National Survey of Children’s Health. SAS Codebook for Data Users: Child and Family Health Measures, National Performance and Outcome Measures, and Subgroups Version 1.0. Data Resource Center for Child and Adolescent Health Supported by Cooperative Agreement U59MC27866 from the U.S. Department of Health and Human Services, Health Resources and Services Administration (HRSA), Maternal and Child Health Bureau (MCHB). www.childhealthdata.org.

[19] United States Census Bureau (2021). National Survey of Children’s Health: Guide to Multi-Year Analysis. https://www2.census.gov/programs-surveys/nsch/technical-documentation/methodology/NSCH-Guide-to-Multi-Year-Estimates.pdf.

[20] SAS Institute, Inc. (2017). User’s Guide: The MI Procedure.

[21] Liljenquist K., O’Neil M.E., Bjornson K.F. (2018). Utilization of physical therapy services during transition for young people with cerebral palsy: A call for improved care into adulthood. Phys. Ther..

[22] Colver A., Rapley T., Parr J.R., McConachie H., Dovey-Pearce G., Le Couteur A., McDonagh J.E., Bennett C., Hislop J., Maniatopoulos G. (2019). Facilitating the Transition of Young People with Long-Term Conditions through Health Services from Childhood to Adulthood: The Transition Research Programme.

[23] Fortuna R.J., Holub A., Turk M.A., Meccarello J., Davidson P.W. (2018). Health conditions, functional status, and healthcare utilization in adults with cerebral palsy. Fam. Pract..

[24] Lariviere-Bastien D., Bell E., Majnemer A., Shevell M., Racine E. (2013). Perspectives of young adults with cerebral palsy on transitioning from pediatric to adult healthcare systems. Semin. Pediatr. Neurol..

[25] Hurvitz E.A., Whitney D.G., Waldron-Perrine B., Ryan D., Haapala H.J., Schmidt M., Gray C., Peterson M.D. (2021). Navigating the pathway to care in adults with cerebral palsy. Front. Neurol..

[26] Pordes E., Gordon J., Sanders L.M., Cohen E. (2018). Models of care delivery for children with medical complexity. Pediatrics.

[27] Kuo D.Z., McAllister J.W., Rossignol L., Turchi R.M., Stille C.J. (2018). Care coordination for children with medical complexity: Whose care is it anyway?. Pediatrics.

[28] Björquist E., Nordmark E., Hallström I. (2015). Living in transition—Experiences of health and well-being and the needs of adolescents with cerebral palsy. Child Care Health Dev..

[29] Center for Disease Control and Prevention, National Center on Birth Defects and Developmental Disabilities Autism and Developmental Disabilities Monitoring (ADDM) Network. https://www.cdc.gov/autism/addm-network/index.html.

[30] Center for Disease Control and Prevention, National Center on Birth Defects and Developmental Disabilities Moving Fragile X Syndrome Research FORWARD. https://www.cdc.gov/fragile-x-syndrome/articles/moving-research-forward.html.

[31] Coyle L.-A., Atkinson S. (2019). Vulnerability as practice in diagnosing multiple conditions. Med. Humanit..

[32] Fauconnier J., Dickinson H.O., Beckung E., Marcelli M., McManus V., Michelsen S.I., Parkes J., Parkinson K.N., Thyen U., Arnaud C. (2009). Participation in life situations of 8–12 year old children with cerebral palsy: Cross sectional European study. BMJ.

[33] Fremion E.J., Dosa N.P. (2019). Spina bifida transition to adult healthcare guidelines. J. Pediatr. Rehabil. Med..

[34] Sarmiento C.A., Wyrwa J.M., Glaros C., Holliman B.D., Brenner L.A. (2024). Experiences of young adults with cerebral palsy in pediatric care transitioning to adult care. Dev. Med. Child Neurol..

